# Examining the capability for rhythmic synchronization and music production in vocal learning parrot species

**DOI:** 10.3389/fpsyg.2023.1271552

**Published:** 2023-11-01

**Authors:** Yoshimasa Seki

**Affiliations:** Department of Psychology, Aichi University, Toyohashi, Japan

**Keywords:** vocalization, synchronization, songbirds, parrots, unison, music, vocal learning, entrainment

## Abstract

Vocal production learning and beat perception and synchronization (BPS) share some common characteristics, which makes the vocal learning and rhythmic synchronization hypothesis (VLH) a reasonable explanation for the evolution of the capability for rhythmic synchronization. However, even in vocal learners, it is rare to see non-human animals demonstrate BPS to human music. Therefore, the first objective of this article is to propose some possible reasons why we do not see BPS in budgerigars, an excellent vocal learning species, while presenting some of my own findings. The second objective of this article is to propose a seamless bridge to connect the capability for vocal learning and BPS in locomotion. For this purpose, I present my own findings, wherein cockatiels spontaneously sang in synchrony with a melody of human music. This behavior can be considered a vocal version of BPS. Therefore, it can establish a connection between these two capabilities. This article agrees with the possibility that some mechanisms other than the vocal learning system may enable BPS, contrary to the original idea of VLH. Nevertheless, it is still reasonable to connect the capability for vocal learning and that for BPS. At the very least, the capability for vocal learning may contribute to the evolution of BPS. From these arguments, this article also proposes a scenario which includes vocalizing in synchrony as a driving force for the evolution of BPS and the capability for music production.

## Introduction

Some forms of music are rendered by a single voice or instrument as solo music, while others are performed by multiple voices or instruments as an ensemble. For the latter type of music, in most cases, synchrony is a key factor. In general, when multiple people synchronize sequential motor outputs, it enables them to accomplish more than one single person could do alone (e.g., pulling a rope in a tug-of-war, rowing a large boat, etc.). During these tasks, people occasionally produce rhythmic acoustic signals with vocalizations, or songs, to coordinate their timing. Therefore, at least in this respect, a capability to produce motor outputs synchronized with rhythmic sound sequences (or, songs) could be adaptive in human evolution, in which cooperating behaviors result in increased fitness or reproductive success.

How about such evolution in non-human animals? Do they possess the capability to move in synchrony with musical rhythms through locomotor and vocal outputs? If so, what are the ultimate and proximate factors? This article addresses these questions by mentioning our own studies that explore the capability for synchronization in non-human animals and by referring to some relevant studies to provide clues for discussing the evolution of rhythmic synchronization.

A distinctive feature of this article is its exploration of vocal output synchronization by a parrot species with melodies of human music. This form of synchronization necessitates the coordination of pitch (frequency), referred to as “spectral synchronization” (Podlipniak, 2023), which is closely related to music production. To facilitate an easier understanding of this type of synchronization, let us visualize sound spectrograms. In most spectrograms, the x-axis represents time, and the y-axis represents frequency. When we overlay one spectrogram onto another and find a match not only along the x-axis but also along the y-axis, we can refer to it as spectral synchronization. This type of synchronization is crucial in the context of choir singing. If a choir member fails to align his or her pitch with that of the other members, it can disrupt the harmony of the performance. Most previous studies examining the capability for synchronization to sound sequences in non-human animals have predominantly focused on tempo or timing, as they often relate to locomotion rather than vocal outputs. Therefore, this article offers a unique perspective that contributes to the discussion of the evolution of rhythmic synchronization in non-human animals.

My main research targets are small parrots. The primary reason for this is that they are excellent vocal learners and can imitate various sounds. In the following section, I will briefly mention this point in connection with the present topic.

## Vocal production learning and rhythmic synchronization

In summary, the main advantage of using parrots in my research is that these birds possess excellent vocal learning capabilities. Vocal production learning results in the addition of novel acoustic patterns into one’s own vocal repertoire based on auditory experience. Historically, it has been suggested that only three groups of birds (passerines, parrots, and hummingbirds) and a limited number of mammal groups (whales, elephants, some small bats, humans, and pinniped species are sometimes included) are capable of vocal learning (Tyack, 2019), while most other animals are limited to producing innate vocal repertoires. Some researchers believe what distinguishes vocal learners from other animals is that they possess developed vocal control systems in the cerebrum which have been well studied, especially in birds and humans (Jarvis, 2004). However, this “all-or-nothing” categorization may not be suitable for this context. For example, some researchers reported vocal convergence in a non-human primate species (Fischer et al., 2020). Further, Petkov and Jarvis (2012) mentioned that the degree of ability varies even among those vocal learners and rated them into several classes, such as high vocal learners (humans), complex vocal learners (e.g., parrots and songbirds), moderate vocal learners (e.g., mice) and limited vocal learners (e.g., monkeys). In addition, we find some anecdotal evidence through videos on the internet describing dogs and cats that can mimic human words. A study reported the Lombard effect is elicited by the brainstem in cats (Nonaka et al., 1997); therefore, those dogs and cats may somehow modify their vocal patterns with an interaction between auditory inputs and motor outputs without such a vocal control system in the cerebral cortex. These phenomena indicate it is not always easy to draw a single line between vocal learners and non-vocal learners. Nevertheless, it is well known that parrots often mimic many sounds, including human words, and most people would accept the claim that parrots are one of the best vocal learning species in the animal kingdom. Thus, I will develop my arguments based on this fact that parrots are vocal production learners.

Similar to vocal production learning, rhythmic synchronization to a musical beat is universally observed in humans. However, there had been no academic literature examining the capability of non-human animals until 2009, when some studies reported that vocal learners possess this capability (Patel et al., 2009; Schachner et al., 2009). There are some similarities between vocal production learning and rhythmic synchronization with sound sequences. (1) Vocal learning requires a real-time comparison of vocal outputs with auditory feedback to verify that the vocalizations are correctly produced. Similarly, rhythmic synchronization requires real-time adjustment of the timing of motor outputs to auditory inputs. Once regular rhythmic patterns are extracted from auditory inputs, it may not always be necessary to adjust the timing of each output to each singular stimulus in real-time to maintain synchronization for the entire sound sequence; however, it is necessary at least in the beginning of synchronization. One study clearly demonstrated that altered auditory feedback resulted in disordered acoustic patterns in the vocalizations of budgerigars, similar to what occurs in humans (Osmanski and Dooling, 2009), thus showing that budgerigars require real-time feedback. Moreover, (2) both capabilities necessitate the transformation of sound inputs into motor outputs (i.e., locomotion or vocalization).

Given the two points mentioned above, it is reasonable that the vocal learning and rhythmic synchronization hypothesis (VLH) was proposed. This hypothesis suggests that entrainment to a musical beat relies on neural circuitry in the brain specialized for complex vocal learning and evolved as a by-product of vocal learning (Patel, 2006; but see Brown, 2022). This hypothesis is supported by an impressive study that demonstrated a Sulphur-crested cockatoo named Snowball moving in synchrony with musical beats (Patel et al., 2009). Therefore, we have good reason to use vocal learning species for synchronization studies.

## Training two bird species to synchronize locomotion with isochronous signals

The capability for beat perception and synchronization (BPS), as predicted by the VLH, enables individuals to align rhythmic movements with the periodicity perceived in complex auditory rhythms, such as music (Patel, 2021). Therefore, BPS differs from synchronization with isochronous auditory sequences, such as metronomic sounds. However, as a first step, studying animals’ ability to synchronize with a metronome may provide insights into more complex forms of synchronization, especially if the subjects can adjust their motor patterns to synchronize with various inter-stimulus intervals. Therefore, my colleagues and I used operant conditioning methods to investigate rhythmic synchronization to isochronous sequences in budgerigars and Bengalese finches. In the finch study, we made improvements to the experimental apparatus, such as the sensitivity of response keys, but the fundamental methods remained consistent across these studies.

**Figure 1:**
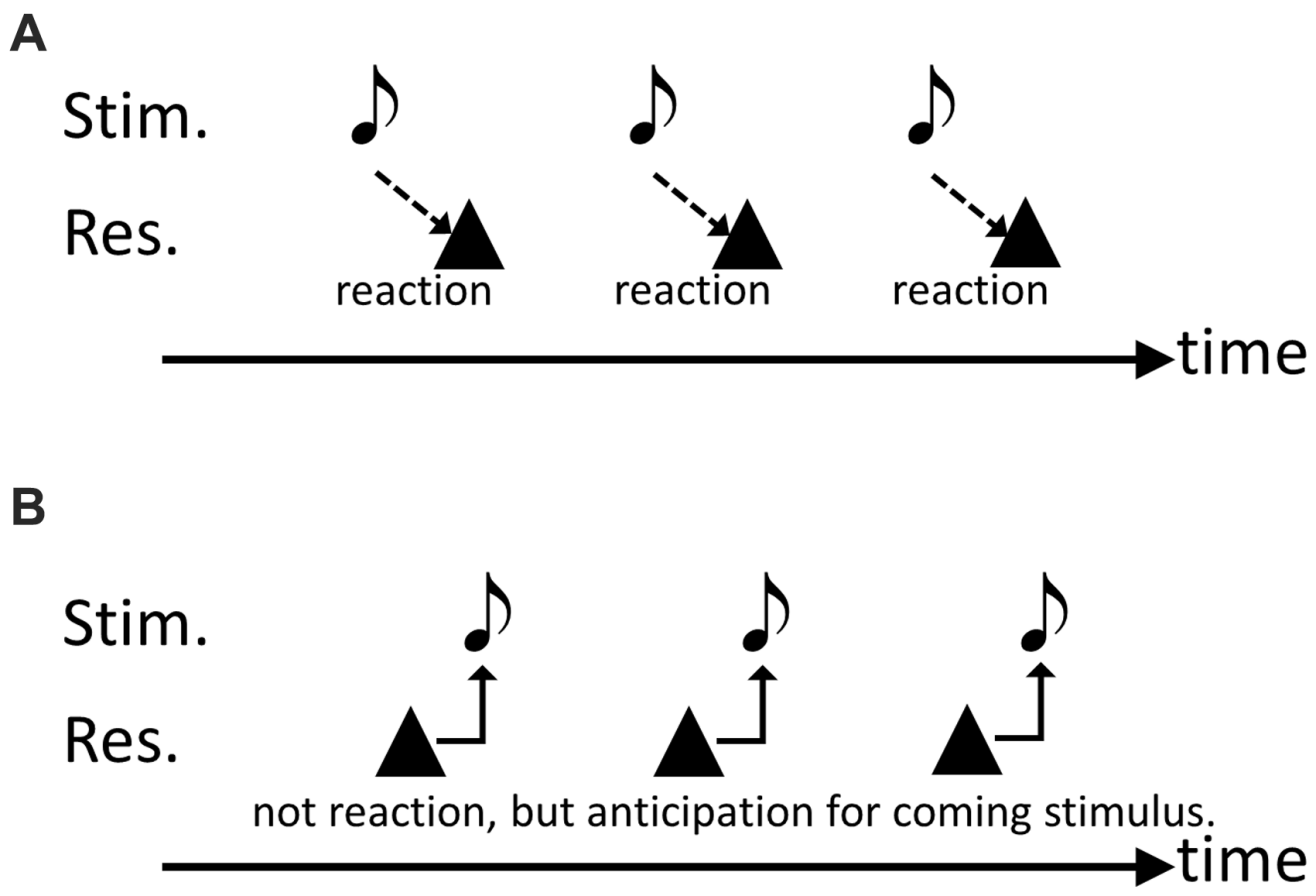
Schematic drawings to explain the possible two strategies in the experiments. **(A)** Responding to each singular stimulus, **(B)** anticipating the timing of the subsequent notes.

First, we examined whether budgerigars could move in synchrony with isochronous audio-visual stimuli (Hasegawa et al., 2011). The birds were successfully trained to continuously peck a key in response to isochronous sound sequences. In this task, the subjects could employ one of two strategies. The first strategy is to move in response to each individual stimulus. In this strategy, the peck response is generated after each stimulus presentation (Figure 1A). The second strategy is to move in anticipation of the timing of the following stimulus. In this strategy, the subjects need to actively discern a rhythm pattern from the rhythmic sequences to anticipate the next stimulus (Figure 1B). The results showed that the average peck response occurred slightly ahead of the stimulus onsets. Therefore, the data suggests that birds employ this second strategy, indicating some degree of anticipation in their behavior (Repp and Su, 2013).

Next, ten Bengalese finches were used as subjects for the same experiment, and the birds were also successfully trained to continuously peck a key in response to isochronous sound sequences. However, in contrast to the results of the budgerigar study, most of them (except one subject) applied the first strategy, where peck timing occurred after stimulus onset. The finches were further trained to produce several pecks in synchrony with metronomic sounds and then two extra pecks without sound stimuli, while maintaining the timing of the metronome. After this training, the finches eventually exhibited a similar trend to the budgerigars, anticipating the timing of the following stimulus (Ikkatai and Seki, 2023). It is worth mentioning that Bengalese finches are “age-limited” or “closed-ended” vocal learners unlike budgerigars which are open-ended learners (Brenowitz and Beecher, 2005), and this factor should be considered for interpretation of the results.

The results of these two studies are not inconsistent with the VLH, but also suggest that the degree of capability for rhythmic synchronization differs between these two vocal learning species. Therefore, the results suggest that capability for vocal learning may not always be directly connected to BPS. This means we should consider other factors, as mentioned later in this article. The story may be further complicated by the fact that birds were rewarded with food. In reality, the studies demonstrated that they can be trained to move in synchrony with isochronous stimuli to obtain a food reward. Therefore, the studies were not demonstrations of spontaneous entrainment to the stimulus sequences, as seen in the Snowball study. Thus, further studies examining whether the birds spontaneously synchronize to rhythmic sound sequences are needed.

## Do rhythmic sound sequences influence locomotion timing without training in budgerigars?

Therefore, I examined whether hearing rhythmic sound sequences affected self-paced key-peck timing in budgerigars (Seki and Tomyta, 2019). The task was to peck two keys, one to the left and one to the right of the bird, alternately and repeatedly. They were rewarded with food much like the experiments mentioned above. However, in this case, they were just trained to peck an illuminated key. Once the key was pecked, the illumination turned off, and the other key was illuminated until pecked. Thus, they created peck sequences at their own pace. Then, metronomic sound sequences were presented during the task, with intervals similar to what the birds were already producing with their self-paced key pecks. One important difference from the experiments mentioned earlier is that the birds were not required to synchronize their peck timing with the timing of the stimulus sounds; in other words, they could ignore the sound stimuli to create their own peck sequences.

Subsequently, we analyzed the interference of the metronomic stimulus sounds on the natural key peck timing. The results showed that the peck timing of some budgerigars was influenced by the sounds. When the peck timing was aligned with the stimulus onset of the sounds, there was a significant bias in the distribution of peck timing. However, a circular statistical test revealed that the bias did not occur around the stimulus onset. This suggests that the locomotion of the budgerigars was affected by the sounds, but they did not have a strong tendency to peck the keys in synchrony with the sounds on their own.

In this task, the driving force behind the birds’ behavior was the desire to receive a food reward. Therefore, they chose the easiest strategy for this purpose, which was pecking the keys regularly and ignoring any external stimuli or factors that could serve as distractions. Furthermore, their locomotion was the result of training and was not voluntary or spontaneous. Other factors, such as social influences, may play a role in timing coordination to rhythmic sequences in birds.

## Timing coordination of trained locomotion between two budgerigars

As described in the first section of this paper, humans occasionally play music with one another, and social factors can influence the formation of this type of behavior. Therefore, we explored whether budgerigars also have the capability to create a series of motor patterns between two individuals.

In a previous study, budgerigars were trained to discriminate the motor patterns of another individual (stimulus bird) for food rewards. The individual’s motor patterns were produced either by pecking or stepping on a key, and the subject budgerigars’ responses were obtained also by either pecking or stepping on a key. The study demonstrated that the locomotion of the subjects was influenced by the locomotion of the stimulus bird, even when this locomotion was presented via video playback (Mui et al., 2008). Therefore, even if we train budgerigars using operant conditioning with a food reward, social factors may positively affect the creation of rhythmic sequences as well. However, we had assumed that it would not be easy to train two individuals to create response sequences in synchrony. Turn-taking differs from synchronization; nevertheless, it is a form of sequential behavior, especially if it is made repetitively. Also, if repetitive turn-taking creates a regular pattern in the tempo and perfect alternation, it is considered antisynchrony, which is closely connected to synchrony (Ravignani, 2015).

Thus, two subjects were placed face-to-face and trained to peck LED keys in turn to create turn-taking sequences (Kishimoto and Seki, 2022). An analysis of peck timing showed that the peck responses were often slightly ahead of LED illumination. Furthermore, the peck timing of a bird was faster when paired with a preferred partner compared to a less preferable partner (the evaluation was conducted through a preference test that measured the duration the bird spent with each partner). Thus, the results suggest that birds did not always use the LEDs as a response cue, but rather they used the visual cue of the partners’ locomotion to create a peck sequence.

Therefore, the results indicate that two budgerigars created action sequences together, and social factors may affect timing coordination in locomotion even in a task for a food reward. This implies that there may be room for improvement in the experiments to detect a potential capability involving rhythmic synchronization in budgerigars using an operant procedure. Typically, while subjects are engaging in an operant task, they are usually kept in a Skinner box and isolated from social factors.

## Why do we not see BPS in budgerigars?

In the above sections, I discussed my attempts to understand the timing coordination of sequential behavior in budgerigars. Taken together, the results of the studies did not reject the VLH. In parallel, the results also did not strongly support the idea that budgerigars have a capability for BPS, even though it is possible that improvements in experimental design could change this, as mentioned above. This is consistent with Schachner et al. (2009). They analyzed hundreds of videos from a large database in which non-human animals danced to music and reported that no budgerigars (0/30 videos) entrained to human music via locomotion.

Therefore, I have listed further possible reasons as to why we have not observed BPS to human music in budgerigars thus far. Please note that this article does not focus solely on the ability of the entire species, but also on the ability of individuals or cohorts, especially on the items (*b*), (*c*), (*d*), and (*e*) below. In humans, we can easily assume that responses to musical sounds can differ greatly between expert musicians and others, with a continuum of responses based on musical experience and talent. Therefore, it is reasonable to consider both the response observed in the entire species and that observed in each individual, which could vary based on their own experiences, especially for individuals kept as domesticated animals.

- Mismatch between body size and time range of beats
  Researchers study beat perception and synchronization in non-human animals using *human music*. The time range of those beats is appropriate for humans to move in synchrony with, given their body size. But they may not be suitable for non-human animals, especially smaller ones.
  In general, smaller animals tend to move more quickly than larger animals. Snowball can move in synchrony with the beats of human music, but keep in mind the body weight of a Sulphur-crested cockatoo is around 800 g. Meanwhile, typical budgerigars weigh only a fraction of that, around 30–40 g. In fact, repetitive movements, such as head bobbing, occur so quickly in budgerigars that humans cannot perform the same movements at the same pace as budgerigars. Further, as shown in Seki and Tomyta (2019), when budgerigars pecked left and right keys alternately at their own pace, they shook their head at a 200 ms interval [i.e., about 300 beats per minute (bpm)]. This is much quicker than the rate at which Snowball danced in synchrony to music (108.7 ± 20% bpm).
  In addition, budgerigars have more fine-grained temporal control of their vocal sounds compared to humans. On a spectrogram, we can see rapid frequency modulation in their contact calls (Farabaugh et al., 1994). Further, when they sing “warble songs,” many notes are produced over a short time (e.g., Tu et al., 2011). Therefore, the comparatively slow pace may make it quite difficult for budgerigars to move in synchrony with beats of human music, even if they are able to extract beats from complex sound sequences (*cf*. Hoeschele et al., 2023). This could be akin to when we hear the songs of humpback whales and perceive the songs to be very slow.
- Variability of the living environment
  Snowball is not a laboratory animal. This bird has been living in a sound-rich environment, which likely provides many chances to listen to various forms of human music. On the other hand, budgerigars used as subjects for psychological experiments are mostly kept in aviaries within a laboratory. They live in a controlled environment and have almost no chance to listen to human music. Therefore, Snowball is much more familiar with human music than typical laboratory birds.
  Small birds often show neophobic tendencies (e.g., Medina-García et al., 2017), which may also affect their reaction to human music. Therefore, it might be important to consider how long subjects are exposed to human music. Possibly, pet birds have an advantage as research subjects in examining active reactions to human music. It should be noted, the results of Schachner et al. (2009) are free from this criticism because most of the videos they analyzed were of pet animals.
- Variability in bonding to human caregivers
  In relation to the above point, laboratory animals are not pets or companion animals. Thus, they usually do not have strong social bonds with their caretakers. It is reasonable to assume that acoustic signals produced by a group member will attract the attention of other members much more than it will attract the attention of an outsider. Animals may consider human music as a social acoustic signal in the same category as human vocal sounds. Thus, the response to human music may differ depending on how bonded the bird is to humans.
- Sex differences
  In budgerigars, both males and females are capable of vocal learning; however, there are sex differences in vocal behavior. For example, the rate of call convergence among male birds is faster than among females (Hile and Striedter, 2000). Both Snowball and Alex (a famous African Grey Parrot that also entrained to musical beats; Schachner et al., 2009) are male birds. Both males and females show rhythmic entrainment in humans, but it has not known whether this capability is sexually dimorphic in parrots. This factor should also be taken into consideration.
- Age and Lifespan
  It is likely that there is a trend to use younger birds in studies using laboratory animals. For example, Hasegawa et al. (2011) used one-year-old budgerigars. In contrast, the age of Snowball was 12 years at the time the study was done (Patel et al., 2009). In addition, the lifespan of parrots is generally long, but differs between species. For example, a paper (Young et al., 2012) mentioned the maximum lifespan of the Sulphur-crested cockatoo is 72.95 years while that of the budgerigar is 18.01 years based on the data provided by the International Species Information System, which has data on wild animal species held in captivity. Therefore, the effects of experience in living with humans can be different between these species, even if experiments are conducted at the same chronological age.
- Emotional state
  In humans, synchronization to musical beats observed by body movements and neural activity depends on several factors, including familiarity with the music and the general attentional state (Kumagai et al., 2018; Park et al., 2021; Weineck et al., 2022). This might also be true for non-human animals, though it is much more difficult to evaluate these factors in non-human subjects.

Of course, it is possible that budgerigars truly do not have the capability for BPS. But before we can conclude this, we must consider the factors above. For this purpose, we can mitigate some of these influences by incorporating control groups in future studies, even though it might be difficult to obtain a sufficient number of subjects to control for some factors.

## What do studies of non-vocal learning species suggest?

One study demonstrated that a sea lion was successfully trained to move in synchrony with human music using an operant conditioning procedure (Cook et al., 2013). Supplementary videos of the study clearly showed the sea lion moving its neck in synchrony with the beat of the melodies. (*Note*: So far, no studies have attempted to train budgerigars to synchronize to beats of human music using operant conditioning methods.) However, this was different from Snowball’s demonstration, as the sea lion was explicitly trained with food rewards, unlike Snowball’s spontaneous response. The behavior of the sea lion might be like humans marching in synchrony with musical beats. When people are required to do so, they can focus on the music and intentionally extract rhythmic patterns from it. Meanwhile, another recent study mentioned that rats spontaneously move their heads in synchrony with the beats of human music (Ito et al., 2022). Similarly, when a person is listening to music, they may unconsciously nod their head in sync with the music’s beat. In such cases, they may not necessarily be consciously focusing on the musical beat; it just happens naturally. Therefore, considering the findings of these two studies, it is important to determine whether the behavior is compelled or voluntary (or spontaneous). Additionally, we should also consider the emotional states of the animals during the behavior. For example, while watching horror movies, we are not likely synchronize our body movements with the background music’s melody. Conversely, the rats mentioned above may have moved their heads in response to a high anxiety situation, as rats were not allowed drinking water for 24 h prior to the experiment and were kept in a bipedal stance. If this assumption is true, behaviors that are similar in appearance may originate from different mechanisms. This idea could be tested through experiments using previous findings and techniques developed in the fields of psychology and neuroscience. For example, anxiety tests using an elevated plus maze, spatial and object exploratory behavior assessments, and measurements of physiological indicators such as skin conductance response and heart rate could be employed. Furthermore, in some cases, when people are listening to music, they may not move in synchrony with the beats. Especially when a novel melody is presented, some people may listen to it very carefully. This does not mean that the person cannot move in synchrony with the beats. This example could also be compared to the behavior of non-human animals. As an anecdote, some acoustic signals, such as unfamiliar melodies, strongly attract the attention of the cockatiels in my laboratory. The cockatiels are likely to focus on the sounds motionlessly and quietly. Therefore, in both birds and humans, the fact that one does not move in synchrony to a rhythm does not necessarily signal a lack of capability. Even if we do not observe rhythmic synchronization in both vocal learners and non-vocal learners, it does not necessarily mean that they are unable to do it. It might simply be a matter of choosing not to do it. Therefore, we need to distinguish between whether they cannot do it or they choose not to do it. Similarly, if we do observe rhythmic synchronization in both types of learners, we must consider whether the origins of their behavior are the same or not.

In the above two studies (Cook et al., 2013; Ito et al., 2022), the authors did not support the VLH. Contrary to the original idea of the VLH, I agree that it is possible that some mechanisms other than vocal learning systems, including the mechanisms proposed by the gradual audiomotor evolution hypothesis (Merchant and Honing, 2014), might be involved in BPS. On some occasions, we see that different neural mechanisms can generate similar behavioral outputs. For example, as described in previous sections, some individual non-vocal learners, such as certain dogs and cats, occasionally modify their vocal sounds so that the acoustic structures become similar to human words. Since these animals are non-vocal learners, these sounds should originate from the nervous system, rather than the vocal control system seen in vocal learners (Tyack, 2019). Another example is that some parrots learn to pronounce a sound similar to /p/ despite their lack of lips (Pepperberg, 2010). The production mechanism of those sounds should differ from ours. This idea could be applied to the given theme as well, which means a similar output (i.e., rhythmic synchronization in locomotion) could be derived from different mechanisms in some species.

Even if non-vocal learners show rhythmic synchronization, Snowball stands out as the most prominent example of spontaneous rhythmic entrainment to human music by a non-human animal. Moreover, as described in the previous section, there are some similarities between vocal production learning and rhythmic synchronization with sound sequences. Therefore, even though the capability for vocal learning is not always necessary to exhibit rhythmic synchronization, it is a very persuasive factor in explaining the origin of BPS. At the very least, it is quite plausible that the capability for vocal learning contributed to the evolution of rhythmic synchronization to a musical beat. The capability for vocal production learning could be involved in more active, spontaneous, and obvious synchronization to a musical beat, as seen in Snowball and humans. Thus, in the next section, I will introduce an idea that will smoothly connect the capability for vocal learning and the capability for BPS.

## Capability for singing in synchrony with music: a bridge connecting the capability for vocal learning and BPS

If BPS is linked to the vocal learning nervous system, it might be more likely that BPS is expressed through vocal behavior rather than locomotor behavior. In other words, if BPS through locomotor production is closely related to the capability for vocal learning, can we also observe BPS through vocal production?

Temporal coordination for singing in unison has been extensively studied in humans (e.g., Palmer et al., 2013; D'Amario et al., 2018). In these studies, the focus was on understanding how humans achieve temporal coordination when singing in unison, rather than questioning their ability to do so. In contrast, when it comes to non-human animals, the question of whether they can achieve this kind of coordination could be an interesting research inquiry.

Some animals vocalize “in synchrony” with others. For instance, in many bird species, there’s a phenomenon known as the dawn chorus, where multiple birds sing “synchronously” at daybreak (e.g., Gil and Llusia, 2020). However, in this context, “synchronously” simply means that multiple birds sing simultaneously at daybreak, without coordinating the timing of each individual note. A similar type of “synchronization” has been documented in sheep, where active behaviors like grazing and walking, as well as inactive behaviors like resting and ruminating, occur together in a group (Gautrais et al., 2007). These examples do not directly relate to the topic at hand.

Another example involves frogs vocalizing in synchrony with each other. In this behavior, frogs adjust the timing of their vocalizations to match others at the individual note level (Greenfield, 1994). While this is interesting, the temporal structure of their vocalizations is essentially a simpler version of isochronous signal sequences. Additionally, the acoustic patterns are not flexible but are innately determined, as frogs are non-vocal learners. A similar argument can be applied to gibbon co-singing. Gibbons’ songs have a complex structure, and they sing these songs in synchrony between mother and daughter (Koda et al., 2013). However, since gibbons are non-vocal learners, the acoustic patterns of their songs are innately determined and constrained by species-specific factors. Therefore, these examples also may not directly relate to the theme of BPS to music.

On the other hand, several studies have reported instances of passerine birds singing in unison. Species like the Forest weaver (Wickler and Seibt, 1980; Gahr et al., 2008), Tropical boubou (Grafe and Bitz, 2004), White-browed sparrow weaver (Voigt et al., 2006), and Neotropical wren (Mann et al., 2006) engage in duets where some parts of their songs are occasionally sung in unison. These findings are intriguing because these birds are vocal learners, meaning that when they sing in unison, they not only synchronize the timing but also the spectral pattern of their song notes, resulting in both temporal and spectral synchronization.

However, it’s important to note that, in general, passerine birds have species-specific acoustic patterns in their songs. As a result, the flexibility to synchronize vocal timing to a sound sequence might be limited in these species. While some passerines, like bullfinches (Nicolai et al., 2014) and starlings (West and King, 1990), have been known to learn and sing melodies from human music, no study has reported such behavior in any passerine birds where they acquire melodies significantly different from their typical song patterns *and* sing them in unison. On the contrary, humans have the ability both to learn and sing a wide variety of melodies and sing them in unison. This distinction sets their capability apart from that of bullfinches and starlings. Now, let us consider parrots, another group of vocal learners. Do they exhibit similar behaviors to humans?

There are many videos on the internet in which cockatiels sing melodies of human music quite well, producing whistle-like or pure tone-like sounds. Such examples include “*The Mickey Mouse Club March*” and “*My neighbor Totoro*.” This behavior is well-known among bird lovers. Furthermore, this behavior has advantages in studies of vocal mimicry by birds. Some researchers may criticize vocal imitation of human words by non-human animals, as it is prone to misinterpretation (e.g., Wynne, 2008; but also see Pepperberg, 2008), but in contrast, the pure tone-like sounds produced by cockatiels result in song melodies that are easily recognizable and can be agreed upon by all listeners.

Therefore, I have studied the singing behavior of cockatiels in association with human music and found that this species can sing human music in synchrony with the playback of the melody (Seki, 2021). The study demonstrated that when a melody was played back, cockatiels spontaneously started to sing in the middle of the playback and synchronized their singing with the rest of the melody (*Note*: the birds had never been trained to vocalize in synchrony because I had never assumed that they would do this). Therefore, this study demonstrated that at least one parrot species possesses both the capabilities for vocal learning and rhythmic synchronization via vocalizations. This could be a direct demonstration that the vocal learning system has an affinity for the capability of rhythmic synchronization. Therefore, the capability for rhythmic synchronization via vocalizations could serve as a bridge connecting the capability for vocal learning and the BPS observed in body locomotion.

In association with this behavior, there is a significant question: Is this behavior observed in the wild, or does it only occur in domesticated environments? Currently, we do not have an answer to this question because there are no academic reports that mention the capability for vocal production learning in wild cockatiels. This species lives in large flocks and frequently covers long distances in the wild, which presents challenges for recording vocalizations from specific individuals in their natural habitat. Therefore, this could be a topic for future studies.

One of the possible psychological substrates of this behavior is automatic imitation. Automatic imitation (and related behaviors) via locomotion by parrots have already been documented (e.g., Mui et al., 2008; Gallup et al., 2015; Ikkatai et al., 2016). Singing in unison might have begun as a vocal version of real-time automatic imitation for learned motor sequences. As an initial step in this behavior, it is possible that the cockatiels might have misinterpreted the auditory input of the playback sounds as the auditory input of their own vocal outputs, causing them to sing in synchrony with the melody. This idea corresponds to a previous finding that songbirds have auditory mirror neurons in the HVC (proper name), a nerve nucleus in the vocal motor pathway of the brain (Prather et al., 2008; HVC corresponds to NLC, the central nucleus of the lateral nidopallium in parrots, Jarvis, 2004). Then, the birds might have discovered that vocalizing in synchrony can attract the attention of human caregivers, and the behavior was reinforced. As I mention in the next section, it is unlikely that one can directly observe evidentiary neural activities for automatic imitation via vocalization in singing cockatiels. Therefore, instead, we may explore which kind of stimulus elicits more singing from cockatiels. For example, if they start singing more frequently in response to sounds that are more similar to their own song, it supports this idea.

To sum up, cockatiels sing human music in synchrony with a playback of the melody. As mentioned in the first paragraph of this section, this finding can serve as a bridge that more smoothly connects the capability for vocal learning and the capability for rhythmic synchronization in locomotion. The cockatiel belongs to the same Cacatuidae family as the Sulphur-crested cockatoo (e.g., White et al., 2011), thus, this provides more relevance when comparing this behavior to Snowball’s behavior. If singing in unison is involved in automatic imitation or auditory mirror neurons, it may imply that those neurons are also somehow (perhaps indirectly) involved in rhythmic synchronization in locomotion, though it is difficult to examine.

## Singing in unison and the evolution of capability for music production

In the VLH, BPS is considered a by-product of vocal learning, meaning that the behavior itself was not adaptive. However, the capability for singing complex songs in unison may increase the fitness of singers. One possible function of this behavior is that it can serve as a means to identify group members and detect outsiders in a population, even if the origin is simply automatic imitation. It should be noted that this idea originates from the fact that one of the functions of imitating contact calls in budgerigars is hypothesized to be group member recognition (Farabaugh et al., 1994, *cf*. Podlipniak, 2023). For example, when people in a group are singing in synchrony, if an outsider sings an incorrect note or word, people can easily detect this individual.

In this context, singing together in synchrony could be considered a cooperative behavior for detecting outsiders. This may help strengthen bonds among group members, akin to synchronization serving as a form of vocal grooming (Dunbar et al., 2012). Singing in synchrony requires coordination of both timing and pitch (in other words, temporal and spectral synchronization), making it more suited to this purpose compared to other behaviors like vocalizing a contact call in synchrony (which does not require continuous temporal coordination) or clapping hands in synchrony (which does not require spectral coordination). This idea could be consistent with the suggestion by Bowling et al. (2013). They examined inter-individual synchronization in vocal production by humans and reported no significant sex differences in timing coordination; thus, they suggested that the origins of musical rhythm might lie in cooperative social interaction rather than sexual selection. In future studies, it would be interesting to compare the ability to produce synchronized motor outputs between vocal and locomotor behaviors, such as hand clapping, and assess the effects of social relationships on these skills, including timing coordination and the creation of regular rhythmic patterns.

Furthermore, the capability for singing in synchrony may have played a role in driving the evolution of the capability for music production in humans, or at least a part of it (e.g., creating and sharing complicated sound sequences with regularity). Even if the origins of this behavior were related to automatic imitation as described above, when a person sings in synchrony with another singer, this behavior might elicit different (and maybe positive) reactions from others [this idea is derived from the phenomenon of singing in unison observed in songbirds. Each individual can sing solo, but sometimes they sing in synchrony with a partner. This appears to be much more effective in inhibiting the behavior of rivals during territorial defense (e.g., Wickler and Seibt, 1980; Grafe and Bitz, 2004)]. Therefore, if such a reaction results in an advantage for the singer, this behavior might be reinforced and become widespread within the group. Subsequently, group members might discover that singing more complicated sound sequences in synchrony serves as a more useful signal for recognizing fellow group members than simpler sequences. As a result, people may develop a preference for and create more complex rhythm patterns. This idea could be tested through a psychological experiment examining whether singing more complicated sequences in synchrony leads to a stronger sense of social bonding than singing simpler sound sequences.

**Figure 2:**
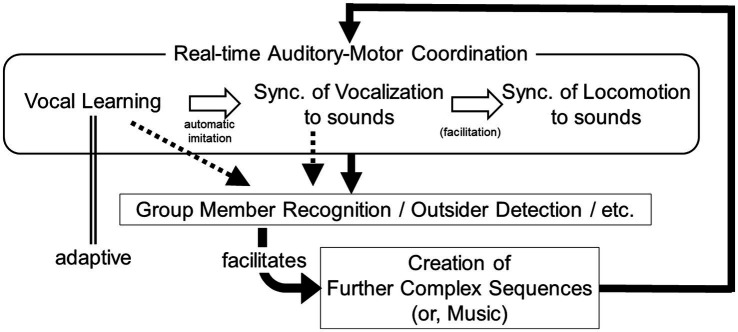
A schematic drawing of the scenario proposed in this article. In this scenario, “synchronization of vocalization to sounds” became the basis of “synchronization of locomotion to sounds” (or, facilitated it). Then, after locomotor synchronization was incorporated, it actively functions to reinforce the loop, in addition to other factors.

Therefore, in this plausible scenario, although the behavior may be influenced by both genetic variations and cultural factors, it could impact the fitness of highly social animals (Figure 2). A similar scenario could be envisioned for non-vocal behaviors like hand clapping and drumming. However, this concept is likely more applicable to vocal behavior, as it can encompass a wider range of variations, necessitating additional manipulation of motor outputs to adjust acoustic parameters like pitch and timbre, in addition to controlling the magnitude and timing of motions.

To the best of my knowledge, no papers have mentioned synchronous singing in parrots in the context of the evolution of music, although some have described synchronous chorusing by other animals within this topic (e.g., Merker, 2000; Ravignani et al., 2014). Nevertheless, considering studies like those involving Snowball (Patel et al., 2009; Keehn et al., 2019), discussing singing in synchrony with human music by cockatiels can be a crucial factor in connecting the capability for vocal learning with rhythmic synchronization in locomotion. This article introduces a new perspective for exploring the relationship between vocal learning and the evolution of BPS.

As another point, sexual selection is often brought up in discussions about the evolution of music (e.g., Miller, 2000). While it is certainly possible that sexual selection played a role in the evolution of human music, there are instances in songbirds where singing together does not appear to be used for mate choice; instead, it is employed for territorial defense (Grafe and Bitz, 2004; also see Fitch, 2006). Additionally, BPS exhibited by the cockatoo and singing in synchrony by cockatiels have been observed in contexts that are not related to mating behavior. Furthermore, as mentioned earlier, these observations align with the results of Bowling et al. (2013). Therefore, I have excluded the concept of sexual selection from this scenario.

A critical component of the scenario is the creation of complex sequences. The cockatiels that sang in unison in my laboratory occasionally created some novel vocal sequences which were shared among them (Seki, 2023). This is just an anecdote, but it is possible that once they learn to sing relatively complicated sound sequences in unison, it may stimulate them to create novel sound sequences (*cf*. Bannan, 2020). Similarly, Snowball created various novel motions while dancing in synchrony with music (Keehn et al., 2019). Therefore, although the scenario for the evolution of capability for the music production in humans is still being worked out and includes many assumptions, it is possible that further experiments in these parrot species may provide some supporting evidence for creation of novel complex sequences.

Here, we can consider one important question: Can we understand the activity of the central nervous system during synchronized movements with rhythmic stimuli in parrots? As a researcher who had utilized several electrophysiological techniques to record neural activities in songbirds, I believe it is not appropriate to apply such methods to parrots (there are many reasons, which I will not discuss here). Instead, in addition to observing their natural behavior, we can use non-invasive techniques to understand the psychological mechanisms. Some researchers have successfully measured the levels of various hormones, such as mesotocin (the homologue of oxytocin in birds; Seguchi et al., 2022; also see Harvey, 2020) and corticosterone (Suzuki et al., 2012) in birds’ urine or fecal matter. This technique, along with recordings of some non-invasive physiological indicators, may help clarify the emotional state during birds’ synchronization to rhythmic stimuli. Additionally, the results may provide clues to determine why some parrots demonstrate synchronization to rhythmic sound sequences and produce vocal and locomotor sequences, while others do not.

In the preceding sections, I have suggested several experiments for future studies. While the methodology may vary between species, including the use of questionnaires for humans and non-invasive physiological methods for parrots, these studies will represent significant steps toward a deeper understanding of the complex interplay of vocal learning, synchronization, and evolutionary fitness. Furthermore, the findings of these studies may offer valuable insights for researchers in applied psychology fields that are related to rhythmic synchronization, such as music education and rehabilitation following accidents or disease.

## Summary and conclusion

The Vocal Learning and Rhythmic Synchronization Hypothesis (VLH) offers valuable insights into the evolution of the capability for beat perception and synchronization with melodies in music. Studies involving two vocal learning species, budgerigars and Bengalese finches, have shown that these birds can be trained to move in synchrony with isochronous sound sequences. However, it’s essential to note that this training does not imply that they consistently and actively synchronize their locomotion to rhythmic sound sequences. Furthermore, there are variations in response patterns between these species, indicating that the capability for vocal learning alone may not be directly linked to beat perception and synchronization (BPS). While these results do not discredit the VLH, they also do not provide strong support for it.

Therefore, as discussed in the section titled “Why do we not see BPS in budgerigars?” it is crucial to consider various factors and conditions that influence the expression of BPS in their behavior. Nevertheless, given the shared characteristics of vocal learning and rhythmic synchronization in locomotion, it remains reasonable to connect these two behaviors. It is plausible that the capability for vocal learning played a role in the evolution of superior rhythmic synchronization in locomotion, even if other mechanisms also contribute to rhythmic synchronization.

To provide a more seamless bridge between these concepts, I introduced findings from my own study involving cockatiels in this narrative. The study demonstrated that when presented with a playback of a musical melody, cockatiels spontaneously joined in singing in synchrony with the melody. Based on this, I proposed a potential scenario for the evolution of the capability for music production, as depicted in Figure 2. While this model primarily applies to humans, evidence for some of its components (e.g., vocal learning, synchronization, creation of novel, and complex sound sequences) can be observed in both humans and parrots, as discussed in previous sections. Consequently, I suggested some empirical experiments to further explore the connections between these components and investigate their functions. These experiments present intriguing challenges for future studies.

## Author contributions

YS: Writing – original draft, Writing – review & editing.
